# Food Intake and Sleep Disorders in Children and Adolescents with Obesity

**DOI:** 10.3390/nu15224736

**Published:** 2023-11-09

**Authors:** Valeria Calcaterra, Virginia Rossi, Veronica Maria Tagi, Paola Baldassarre, Roberta Grazi, Silvia Taranto, Gianvincenzo Zuccotti

**Affiliations:** 1 Department of Internal Medicine and Therapeutics, University of Pavia, 27100 Pavia, Italy; 2 Pediatric Department, Buzzi Children’s Hospital, 20154 Milano, Italy; virginia.rossi@unimi.it (V.R.); veronica.tagi@unimi.it (V.M.T.); paola.baldassarre@unimi.it (P.B.); roberta.grazi@unimi.it (R.G.); silvia.taranto@unimi.it (S.T.); gianvincenzo.zuccotti@unimi.it (G.Z.); 3 Department of Biomedical and Clinical Science, University of Milano, 20157 Milano, Italy

**Keywords:** pediatric obesity, sleep disorders, children, adolescents, food intake, diet

## Abstract

Over the last few decades, numerous scientific studies have investigated the possible association between sleep duration and adiposity during childhood, since it has been reported that sleep deprivation causes a related increase in caloric intake. Even though the underlying pathogenetic mechanisms are still under study and not completely known, the effect of dietetic habits and nutrient intake on sleep quality and patterns has been reported. The aim of this study is to explore the intricate interplay between food intake/diet patterns and pediatric sleep disturbances in children and adolescents with obesity, emphasizing the importance of not underestimating this aspect in the prevention and treatment of this complex disease. Recent evidence supports a high correlation between specific diet patterns and foods with sleep disturbances in children at all ages. Diets rich in fiber, fruit, vegetables, and anti-inflammatory nutrients and low in saturated fats seem to promote better sleep quality. Sleep disturbances are, in turn, risk factors for the development of obesity. Therefore, food strategies should be applied to counteract this harmful process. Unraveling the complex links between dietary habits, sleep patterns, and obesity is essential for developing effective strategies to combat this critical public health issue.

## 1. Introduction

Childhood and adolescent obesity represent a serious and escalating global health concern with continuous growth in their prevalence. The disease’s prevalence increased from 0.7% to 5.6% for girls and from 0.9% to 7.8% for boys aged 5–19 from 1975 to 2016; in recent years, with the impact of the COVID-19 pandemic, weight gain in children has further increased [1,2]. Obesity is characterized by an abnormal accumulation of adipose tissue that impairs health. The etiology of essential obesity is multifactorial, including unhealthy eating patterns and a sedentary lifestyle, genetic and epigenetic influences, and environmental factors, and the determinants of obesity act synergistically. As is already known, it is associated with many multi-organ complications that, if not adequately recognized and treated, persist into adulthood, leading to increased morbidity and mortality [3,4]. 

Over the past few decades, a plethora of scientific works have investigated the potential connection between sleep duration and adiposity during childhood. This research has been prompted by findings indicating that sleep deprivation is linked to a significant increase in caloric intake [5]. However, underlying pathogenetic mechanisms are still under study and are not completely known. Sleep not only has a restorative function but also plays a central role in regulating metabolism, influencing appetite control, and affecting immunological functions. Acknowledging the importance of sleep, reputable organizations such as the American Association of Pediatrics (AAP) and experts from the National Sleep Foundation have released consensus recommendations outlining the appropriate daily sleep duration for children in various age groups [6,7].

Among sleep disturbances, obstructive sleep apnea syndrome (OSAS) represents the most common form of sleep disturbance in obesity, which consists of prolonged respiratory pauses altering the child’s sleep patterns. Obesity also leads to hormonal imbalances, particularly insulin resistance, which can disrupt sleep–wake cycles and the appetite–satiety circuit. Key hormones, such as insulin, leptin, ghrelin, and cortisol, as well as orexins, hypocretins, and melatonin, are believed to play a crucial role in the complex relationship between sleep and obesity [8,9,10]. Additionally, the excess body fat present in children with obesity often results in physical discomfort, including skeletal pain, and psychological issues like poor self-esteem, anxiety, and depression, all of which can negatively impact their sleep quality. Children with obesity or who are overweight often complain of insomnia, with trouble falling asleep or staying asleep at night, leading to daytime sleepiness and fatigue. These sleep-related issues, in turn, affect their attention and concentration, thus influencing their school performance, behavior, and overall quality of life [11]. 

Sleep quality and patterns may be significantly influenced by dietetic habits and nutrient intake. In particular, a high-protein meal or the excessive consumption of fatty foods before bedtime or late-night snacking on sugary foods are related to poor-quality sleep. In contrast, a meal containing healthy fats, such as omega-3, may have beneficial effects on sleep. Furthermore, eating a well-balanced diet that includes a variety of nutrients, like vitamins and minerals, found in fruits and vegetables, can help to improve one’s quality of sleep and overall well-being [12,13]. 

Addressing the connection between childhood obesity and sleep issues requires a holistic approach involving parents, pediatricians, and educators. The aim of this approach is to promote healthy nutrition, encouraging physical activity and establishing hygiene sleep practices through a regular sleep schedule and a comfortable sleep environment. Ideally, understanding different features of sleep (duration, timing, and chronotype) may contribute to developing new sleep-focused interventions to prevent obesity. 

The aim of this study is to explore the intricate interplay between food intake/diet patterns and pediatric sleep disturbances in children and adolescents with obesity. It underscores the significance of recognizing and addressing this aspect in the prevention and treatment of this complex disease. Practical implications and research directions are included in the last section to provide a more comprehensive understanding.

## 2. Methods 

We conducted a narrative review to explore the link between dietary patterns and sleep disorders in pediatric populations with obesity. We conducted an extensive literature search using the PubMed and Scopus databases, with the language restricted to English only and including publications from the past 20 years. 

We included all types of articles in our review: original research articles, reviews, meta-analyses, case series, clinical practice guidelines, and commentaries, all pertaining to children with obesity aged 1–18 years who experienced sleep disturbances, encompassing disorders of initiating and maintaining sleep, disorders of excessive somnolence, disorders of sleep–wake schedule, and dysfunctions associated with sleep, sleep stages, or partial arousals (parasomnias or obstructive sleep apnea). 

The list of keywords used and the number of articles found and analyzed for each paragraph are summarized in Table 1. Starting from a total of 699 papers, the authors revised the abstracts of the available literature (n = 313) and reviewed the full texts of the relevant articles (n = 93; 64 original research articles, 11 meta-analyses, 15 reviews, and 3 guidelines) which were analyzed to provide a critical discussion. Additionally, the reference list of all articles was checked.

**Figure 1:**
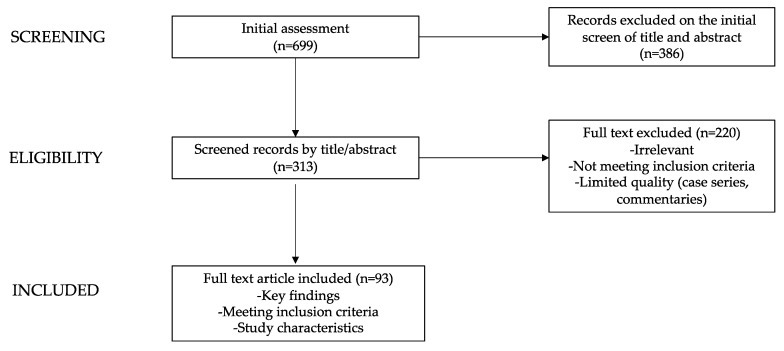
Flow chart showing the process of paper selection and exclusion.

In Figure 1, a diagram graphically showing the process of paper selection and exclusion is reported.

## 3. Sleep Disturbances and Their Connection to Pediatric Obesity

### 3.1. Sleep and Overall Well-Being

Sleep assumes a fundamental role in safeguarding our physical and mental well-being, and its profound impact on the body’s intricate functions cannot be overstated. The imperative functions included in sleep include the regulation of hormonal balance, metabolism, and immune system activity, and the sustenance of our psychological and cognitive faculties. This underscores the critical importance of sleep in maintaining overall health and improving quality of life [10]. 

Recognizing the paramount importance of sleep, the AAP has issued comprehensive guidelines regarding the recommended daily sleep durations for children of various age groups [7]. According to the AAP, children aged 3–5 years, 6–12 years, and those older than 12 years should target 10–13 h, 9–12 h, and 8–10 h of sleep, respectively, including naps [7]. It is worth noting that sleep requirements can slightly vary during different developmental stages in children and adolescents. Interestingly, while the AAP provides these guidelines, experts from the National Sleep Foundation offer slightly differing recommendations. They propose 9–10 h of sleep per night for children aged 6–13 and suggest that adolescents aged 14–17 aim for 8–10 h of sleep [6]. These variations emphasize the nuanced nature of sleep needs, taking into account factors like age and individual biological and physiological differences, including sex and gender differences. Indeed, as Mallampalli et al. [14] have reported, sex chromosomes and the gonadal hormones are the primary contributors to variances at the cellular, organ, and systemic levels between females and males. Additionally, a combination of environmental, cultural, social, and biological factors influences gender differences. 

These guidelines recognize the variations in sleep needs during different developmental stages, ensuring that children receive the adequate amount of sleep necessary for their physical and cognitive growth [7]. 

In the field of sleep research and evaluation, a structured sleep framework includes four basic dimensions [15]: sleep duration, sleep quality, sleep efficiency, and sleep timing.

“Sleep duration” quantifies the amount or length of time spent asleep. This dimension is critically important because it underlies our holistic well-being: adequate sleep duration is essential for maintaining overall health and vitality.

“Sleep quality” can be objectively assessed by examining the architecture of sleep, including the adequacy of time spent in different sleep–wake cycles, or subjectively evaluated based on one’s satisfaction with sleep or perceived sleep-related issues. It also serves as a multidimensional tool, allowing us to measure both quantitative and qualitative aspects of our sleep. 

“Sleep efficiency” measures sleep continuity, taking into consideration the ease of initiating sleep (sleep latency) and maintaining it (minimizing wake episodes). Alternatively, it can be represented as the percentage of sleep time achieved between going to bed and waking up. This dimension plays a key role in determining how restorative our sleep really is. 

Finally, “sleep timing” concerns the temporal aspects of our sleep patterns. It involves determining the specific times at which we go to bed and wake up in a 24-h cycle. These times may vary according to individual preferences and daily obligations. The crucial aspect is to align sleep times with natural circadian rhythms and the demands of daily routine. Doing so maximizes the benefits derived from the restorative nature of our sleep [15].

The comprehension and assessment of sleep through the delineated dimensions, including duration, quality, efficiency, and timing, equip individuals with insights into optimizing their sleep patterns for the betterment of their overall well-being.

### 3.2. Determinants in Disordered Sleep and Obesity

Numerous studies have highlighted the adverse impact of sleep deprivation on both mental and physical health. It can impair cognitive functions, disrupt hunger regulation, weaken the immune system, and contribute to metabolic disorders. 

Epidemiological studies consistently show that insufficient sleep, typically defined as 6 to 7 h per night, is associated with a higher risk of metabolic and cardiovascular diseases, including obesity [16,17,18,19].

Sleep disorders have emerged as a modifiable lifestyle factor linked to an increased risk of obesity, especially in children. While establishing causation remains challenging, the existing literature highlights significant correlations involved in this link. Indeed, various aspects of sleep patterns are correlated with factors that influence obesity risk, including appetite and hunger control, hormones, physical activity, and genetic factors. These connections suggest that the relationship between sleep and obesity may be mediated through the regulation of these factors [20,21,22].

The lateral hypothalamic area and related endocrine function are crucially involved in motivated behaviors and regulate the sleep–wake cycle, food intake, and energy balance. It houses neurons that produce hypocretin, which influences sleep, appetite, and energy metabolism. When glucose levels decrease, hypocretin neurons become active, increasing food intake. Conversely, rising leptin levels inhibit these neurons, reducing food intake. Sleep deprivation can enhance hypocretin activity, leading to increased wakefulness and appetite. These neurons boost alertness and physical activity while reducing sleep, and sleep deprivation weakens their ability to promote physical activity and energy expenditure [10].

Research among adults indicates that sleep deprivation can influence hormones regulating appetite, potentially reducing leptin levels and increasing ghrelin concentrations, leading to heightened hunger sensations [23]. Furthermore, one study, the Kiel Obesity Prevention Study, observed elevated leptin levels in girls with sleep restriction, suggesting that chronic sleep loss may lead to future metabolic alterations [24]. Controlled experiments have demonstrated that repeated episodes of partial sleep deprivation (4 h in bed) can trigger hormonal changes, including notable shifts in leptin, ghrelin, and the hypothalamic–pituitary–adrenal axis, potentially compromising glucose tolerance and increasing autonomic activity [25]. Following a “recovery” sleep period with ample rest (12 h in bed), various metabolic and endocrine parameters appear to return to a more favorable functional state [25].

Kim et al. linked compromised sleep quality and disrupted sleep cycles to insulin resistance, disturbances in appetite hormones like leptin and ghrelin, reduced melatonin levels, and metabolic issues. Studies on sleep restriction in adults have shown that changes in hormones, particularly leptin, can lead to increased calorie intake, impacting weight outcomes [26]. Furthermore, disturbances in melatonin levels have been linked to feelings of daytime drowsiness, reducing physical activity levels and increasing the risk of obesity during both childhood and adulthood [27,28].

Inadequate or fragmented sleep can also affect glucose metabolism and the secretion of hormones such as growth hormone, prolactin, glucocorticoids, catecholamines, and testosterone [10,29,30,31]. 

Dietetic habits may have an impact on sleep quality [32,33], resulting from unhealthy fluctuations in sleep-related hormones. The high consumption of processed and sugary foods can lead to increased insulin and leptin levels, potentially disrupting behavioral and circadian cycles, resulting in irregular sleep–wake patterns [34]. High-carbohydrate intake may also stimulate ghrelin, which plays a role in sleep and appetite quality [35].

Nutrient intake may also influence sleep via the regulation of serotonin and melatonin synthesis [36]. Tryptophan is an essential amino acid and precursor of serotonin and melatonin, which are related to sleep and alertness. Therefore, foods that interfere with tryptophan availability and serotonin/melatonin synthesis may impair sleep regulation [36]. 

Physical activity is considered as an additional determinant involved in the complicated diet–sleep–obesity nexus [8]. Irregular sleep patterns and inadequate sleep duration can disrupt daily physical activity levels, promoting sedentary behavior and elevating the risk of obesity. Irregular sleep and insufficient nocturnal sleep can disrupt daily activity rhythms, increase fatigue, hinder physical activity engagement, and foster a sedentary lifestyle, further elevating the risk of obesity [37].

Reduced sleep duration has been linked to heightened inactivity, as the weariness stemming from insufficient sleep amplifies sluggishness and impacts an individual’s overall emotional state [38,39,40]. Additionally, quicker sleep onset has been observed in children engaging in higher levels of physical activity throughout the day [41]. It is logical to contemplate that sleep deficiency and the resulting fatigue could influence physical activity levels in children. Nevertheless, there is limited empirical proof to suggest that disrupted sleep has an effect on any facet of energy expenditure [42]. 

Furthermore, genetic factors significantly influence both obesity and sleep duration [43,44]. 

Finally, sustained sleep disruption in childhood can trigger inflammatory responses similar to those related to obesity [45]. The two disorders may amplify each other and act synergistically, increasing the severity of their respective adverse consequences [46].

## 4. Sleep Disorders in Children and Adolescents with Obesity

Childhood obesity and inadequate sleep have a reciprocal relationship. Shortened sleep duration is a risk factor for developing and sustaining childhood obesity. A higher prevalence of sleep disorders, including breathing disorders related to sleep such as OSAS [47,48], has also been described.

### 4.1. Sleep Deprivation in Pediatric Obesity

Numerous epidemiological studies have scrutinized the correlation between sleep duration, predominantly nocturnal sleep, and weight status in children and adolescents. While these investigations cannot establish direct causation, they provide pivotal insights into the role of sleep in the ongoing obesity epidemic. 

Dimensions of sleep, such as sleep quality, efficiency, and timing, can also independently influence the sleep–obesity association, though there is comparatively less research on them [15].

Snell et al. [49] discovered that for each additional hour of sleep obtained during childhood (between the ages of 3 and 12), there was a 0.75 kg/m² decrease in caregiver-reported BMI at a later time (when the children were between 8 and 18 years old). Similarly, Touchette et al. [50] identified that children who consistently slept very little between the ages of 2.5 and 6 were 2.9 times more likely to present with overweight status or obesity compared to children who consistently slept 11 or more hours per night, even after accounting for potential confounding factors. 

Each additional hour of sleep obtained during childhood (on average, between ages 5 and 11) corresponded to a 35 percent reduced risk of obesity at age 32 [24].

Multiple scientific studies have also shown that increased sleep duration is associated with reduced body fat. For instance, 7-year-olds who slept 9 or more hours per night had 3.34 percent lower body fat (compared to those who slept less than 9 h per night) [51], with gender-specific differences in some instances. Reduced sleep duration (<8 h per night) was negatively associated with total body fat and truncal fat, but positively associated with lean mass percent only in girls [52]. Moreover, a significant association between sleep duration and waist circumference was observed exclusively in girls in this study [52]. In contrast, reduced sleep duration was linked to increased free fat mass in both sexes, but differences in body fat percentage were observed mainly in girls [53]. Additionally, when evaluating waist circumference values, only girls who had shorter sleep durations exhibited significantly larger waist circumferences; on the contrary, a longer sleep duration was associated with reduced waist circumference only in boys [54].

Bedtime seems to be a crucial factor influencing weight status among school children. There is stronger evidence indicating that later bedtimes are associated with increased weight status compared to later wake times. This observation may be primarily attributed to the widespread adherence to a fixed school routine, which tends to regulate morning wake times, while bedtime routines tend to vary more among individuals within this target population [55,56].

Studies suggest considering the bedtime and sleep midpoint, apart from sleep duration, in the sleep–obesity link due to negative behaviors associated with delayed sleep [57,58,59,60], such as the widespread use of electronic devices like tablets, mobile phones, computers, and television. The blue light emitted by electronic devices, particularly when used at night, can lead to a decrease in melatonin release and consequently delay the initiation of sleep. This disruption in the sleep–wake cycle may ultimately contribute to disturbances in circadian rhythm [37].

As a result, delayed sleep timing not only reduces the duration of restorative sleep but also provides more time for behaviors that contribute to obesity [58,61].

According to Bagley et al. [62], the length of wake episodes seems to have more influence on BMI than the number of episodes. Indeed, short episodes also represent an opportunity for bedtime snacking [62].

As described, a later bedtime has been positively associated with an increased risk of overweight status or obesity, whereas no significant association has been found between the time of waking up and the risk of obesity [63]. However, an increased sleep duration during weekends (school terms) and vacations alone is partly sufficient to mitigate the risk of obesity in children [25].

### 4.2. Sleep-Disordered Breathing in Pediatric Obesity

Children and adolescents who are overweight or obese are more affected by breathing disorders related to sleep than their peers [47,48]. Indeed, the prevalence of breathing disorders is 33% to 61% in obese youths in contrast to 1% to 3% in the general pediatric population [64,65].

Sleep-disordered breathing (SDB) refers to a group of disorders characterized by abnormalities of respiration or ventilation during sleep. It encompasses OSAS, the most common in children with obesity, central sleep apnea (CSA) syndromes, sleep-related hypoventilation, and sleep-related hypoxemia disorders [66].

OSAS, mainly caused by enlarged tonsils and adenoids but also exacerbated by obesity, is considered one of the most severe co-morbidities associated with childhood obesity. There is a significant correlation between OSAS and markers of metabolic syndrome (insulin resistance, dyslipidemia, and higher leptin levels), as well as a strong relationship between visceral adiposity and OSAS independent of BMI [67]. Indeed, two mechanisms have emerged explaining the correlation between obesity and OSAS: the increased presence of fat at the level of the pharyngeal soft tissue reducing the caliber of the lumen, and excessive fat in the thoracic and abdominal walls reducing respiratory function [68]. 

OSAS involves upper airway blockages during sleep, leading to reduced oxygen levels and episodes of elevated carbon dioxide levels, followed by awakenings and rapid reoxygenation. Frequent apneas disrupt sleep, causing shifts from deep sleep to lighter stages or even wakefulness [69,70]. 

Research has shown that children with obesity often exhibit a connection between reduced rapid eye movement (REM) sleep, shorter overall sleep duration, and OSAS. Additionally, REM sleep is linked to variations in the appetite-regulating hormone leptin while sleeping. There is a hypothesis that shorter REM sleep might lead to heightened appetite due to elevated leptin levels [71,72].

Children with obesity exhibited notably higher occurrences of SBD, such as obstructive apneas, hypopneas, and episodes of oxygen desaturation per hour of sleep when compared to their peers of normal weight [73].

In the literature, the association between SDB and insulin resistance is well-documented, and it is attributed to various mechanisms [64,74,75]. First of all, sympathetic overactivity, due to intermittent hypoxia and sleep fragmentation, leads to the release of catecholamines, decreased glucose absorption by insulin, and insulin resistance. Additionally, SDB-related factors such as intermittent hypoxia can promote gluconeogenesis and disrupt glucose-induced pancreatic β-cell function, further contributing to insulin resistance [64,74,75]. 

Therefore, the combination of SDB and pediatric obesity contributes to systemic inflammation and elevates the risk of cardiovascular and metabolic diseases [76,77]. 

Since sleep-disordered breathing in children with obesity often goes unnoticed by parents, the assessment of childhood obesity should routinely include screening for sleep disorders with the use of polysomnography. Indeed, it is crucial to emphasize the significance of addressing SDB, particularly in obese adolescents, in order to reduce its adverse effects on cardiometabolic health.

## 5. Dietary Patterns and Sleep Disorders in Pediatric Obesity 

The relationship between sleep quality and dietary preferences is a critical aspect of this discussion. 

While the impact of sleep duration and quality on obesity in children has been well explored in the literature, less information is available regarding the effects of diet on sleep. Indeed, recent studies suggest the presence of a causative correlation between specific dietary patterns and sleep [13]. 

Not only the quantity but also the quality of macronutrients might play a role in the pathogenesis of sleep disorders in children with obesity. Indeed, it seems to worsen sleep quality [78]. Furthermore, a higher consumption of saturated fats has been shown to have a more negative effect on sleep than the higher consumption of polyunsaturated fats [74]. 

The intake of macronutrients plays a pivotal role in the diet–sleep connection. Inadequate sleep may lead to the consumption of foods of lower nutritional quality, characterized by higher fat content [78], more processing, a higher simple–complex carbohydrate intake proportion, and an elevated glycemic index [37]. 

It has been demonstrated that the total duration of sleep and the duration of slow wave sleep (SWS) and REM sleep, registered by polysomnography, are shorter in consumers of high-fat and high-carbohydrate diets [79,80,81,82]. High snack consumption and low protein intake have also been associated with short sleep [83,84]. 

Remarkably, low protein intake (<16% of total energy intake) has been associated with difficulties in initiating sleep, while high protein intake (>19% of total energy intake) may lead to difficulties in maintaining sleep [37]. Sugary foods show a negative association with sleep quality, and reduced sleep hours are related to the increased consumption of sugary beverages and sweets in general [37]. 

Beverage consumption may represent another determinant of poor sleep duration, both as a source of added sugars [85] and because regular energy drink consumption is an independent risk factor for insufficient sleep, as recently demonstrated in a group of 1287 adolescent students in Belgrade [86]. 

In addition, the later timing of meals seems to contribute to later bedtimes and shorter sleep durations in children [87].

As reported, maintaining an early bedtime routine is linked to improved diet quality, characterized by higher fruit and vegetable consumption [88]. Conversely, individuals who habitually go to bed late tend to consume a higher quantity of poor-quality foods. In the context of the Kiel Obesity Prevention Study, the researchers identified a clear trend. Children with shorter sleep durations (less than 9 h per night) exhibited a greater inclination towards the increased consumption of fast food and soft drinks [53]. Food-insecure children grappling with sleep difficulties showed heightened intake of soft drinks, emphasizing the intricate interplay between dietary patterns and sleep challenges [89]. Na et al. [90] added further depth to this connection, revealing that food insecurity was closely associated with compromised sleep quality. The investigations conducted by Jong et al. [91] delved into determinants of short sleep duration, uncovering age- and gender-dependent eating habits that contribute to this issue. In a general sense, the consumption of sugary foods, the absence of a consistent eating routine, and the habit of watching TV during meals were linked to shorter sleep durations. A notable 2.6-fold increase in the risk of sleep disturbances and a subsequent impact on sleep quality is associated with nighttime consumption of stimulant beverages, such as chocolate milk, soft drinks, coffee, and black tea [92]. Furthermore, individuals experiencing more daytime sleep and less nighttime sleep reported stronger food cravings [93]. A noteworthy connection between shorter sleep durations and the heightened consumption of energy-dense foods, including items like pizza and pasta and those containing refined sugars, was also uncovered. This relationship was particularly pronounced in boys and during weekdays [94].

The structure of family meals may also influence sleep habits; eating at fast food restaurants or in front of the TV was associated with poorer sleep quality [95].

Dietary habits may have different effects depending on age. Some studies have explored the association between dietary patterns and sleep in different age groups, with regard to school-aged children in particular. 

The literature data support the association between total energy intake with the risk of developing sleep breathing disorders and sleep–wake transition disorders [12]. It is still unknown as to whether the time of the consumption of the last meal may contribute to worsened sleep duration and quality [12,96]. Moreover, shorter sleep duration in children aged 7 to 11 has been associated with the higher consumption of fast foods and sweet processed snacks [38,94,97,98]. Additionally shorter overnight sleep duration in 6–9-year-old girls [53,99] and 6–13-year-old boys [53] was associated with the higher intake of soda regardless of its caffeine content. In adolescence, shorter sleep duration and greater variability in night-to-night sleep duration were found to be related to the increased intake of soda [97,100].

Ramírez-Contreras et al. [101] showed that short sleep duration and greater sleep disturbances are significantly related to what and how school-aged children eat. In fact, they found that the daily consumption of sweets and candy as well as eating pasta or rice five or more times per week were two dietary habits associated with shorter sleep duration. Furthermore, they observed that later sleep–wake schedules were significant predictors of poor diet quality. In particular, in their study, subjects with a later sleep pattern were more prone to skipping breakfast. Although children with a late bedtime usually prefer to skip breakfast for extra sleep time [102], this behavior should not be encouraged, given the known association between breakfast skipping, obesity, and poor sleep quality [102,103,104]. Finally, this study supported the association between short sleep duration, sleep disturbances, and higher BMI in children [101]. 

Regarding pre-school-aged children, available data on the link between diet and sleep are scarce but in line with reports on the school-aged population. Greater social jet lag (the difference between the weekday and weekend mid-sleep point) in children aged 3–5 has been associated with the higher consumption of fried, processed foods [33]. Furthermore, a longitudinal study suggested a correlation between shorter sleep durations (<10 h) at 2, 3, and 5 years of age with the more frequent consumption of processed and fast foods at age 2 [105]. An high intake of healthy foods does not seem to guarantee higher sleep quality itself, but rather, the more frequent consumption of fast food and soda resulted more strongly in issues related to poorer sleep outcomes [85]. Interestingly, parenting factors, such as using more relaxed discipline strategies, do not seem to influence sleep quality directly but could impair dietary habits. Therefore, caregivers should be educated to encourage healthier dietary choices in order to contribute to better sleep quality in children. Further studies are needed to understand the association between the timing of meals or consistency in household schedules and sleep duration and quality [85].

Focusing on diet and sleep in children in their early life, it has been showed that children with high soft drink, snack, and fast food intake had increased odds of inadequate sleep and frequent night waking [106]. High dairy intake was also associated with night waking. Moderate and high vegetable intake and moderate meat, meat alternatives, or egg intake were associated with decreased odds of frequent night waking [106]. In line with the study by Holmes et al. [85], parents’ use of food to soothe children in distress was also demonstrated to impact dietary intake and sleep [107]. Regarding maternal diet, maternal alcohol consumption before and 9 months after birth was associated with decreased odds of night waking and inadequate sleep, respectively, suggesting that early alcohol exposure may impact sleep patterns in the long term [106].

Although further studies are needed to confirm the ability of certain foods and food patterns to improve sleep, on the basis of the scientific evidence available, some possible nutritional advice for children with obesity and sleep disturbances could be to reduce sugar and fat intake and ensure adequate daily intake of proteins rich in tryptophan, fibers, melatonin-rich foods, and foods rich in folate and antioxidants. In addition to folate, other B vitamins, such as vitamin B12, could also affect sleep quality [108,109,110]. In general, diets rich in fiber, fruit, vegetables, and anti-inflammatory nutrients and lower in saturated fats (e.g., the Mediterranean diet) seem to promote better sleep quality [78]. 

## 6. Conclusions

**Figure 2:**
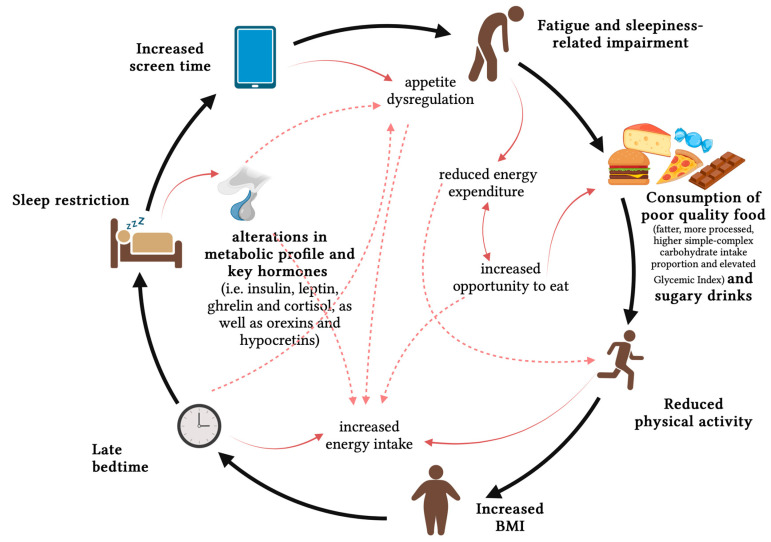
The interplay between sleep and pediatric obesity: investigating the connecting factors (created on https://app.biorender.com accessed on 30 September 2023).

The connection between food consumption, sleep disorders, and obesity in children and adolescents is multifaceted and intricate (Figure 2). 

Diets rich in fiber, fruit, vegetables, and anti-inflammatory nutrients and lower in saturated fats seem to promote better sleep quality. Sleep disturbances are in turn risk factors for the development of obesity. Biological, psychological, and physiological factors may underlie the association between dietary habits, sleep patterns, and obesity. 

The implications of the diet–sleep–obesity relationship for healthcare professionals are profound. Physicians, psychologists, and dietitians must recognize the potential impact of sleep disturbances and dietary choices on the health of children and adolescents with obesity. Early identification and intervention are crucial for promoting healthier eating habits and sleep patterns [63]. 

Improving sleep quality may serve as an alternative strategy to prevent and reduce pediatric obesity by promoting healthier food choices [37].

Future research should delve deeper into this intricate relationship, employing larger sample sizes and objective measures of sleep and food consumption to gain a more comprehensive understanding of this association [37]. 

## Figures and Tables

**Table 1:** List of keywords and the number of articles found and analyzed for each paragraph.

Paragraph	Keywords	Total Number of Articles Found	Relevant Articles (Fully Read)
Sleep disturbances in pediatric obesity	“pediatric obesity” AND “sleep” OR “sleep disorders” OR “sleep apnea syndrome”	242	133 (36)
Diet and Sleep	“children” OR “adolescents” AND “sleep” AND “diet” OR “food”	214	83 (25)
Food intake and sleep disorders in children and adolescents with obesity	“pediatric obesity” AND “food intake” AND “sleep” OR “sleep disorders”	243	97 (32)
